# The impact of the digital economy on urban total factor productivity: mechanisms and spatial spillover effects

**DOI:** 10.1038/s41598-023-49915-3

**Published:** 2024-01-03

**Authors:** Shaohui Zou, Zhe Liao, Xiangbo Fan

**Affiliations:** School of Management, Xi’an University of Science and Technology, 58 Yantazhong Street, Xi’an, 710054 China

**Keywords:** Environmental social sciences, Energy science and technology, Mathematics and computing

## Abstract

Digital economy is the indispensable pathway for driving industrial structural upgrading and fostering innovation and entrepreneurship in cities, ultimately facilitating China's economic transformation. Simultaneously, the enhancement of urban total factor productivity (TFP) serves as a crucial means to achieve high-quality economic development in cities. This study examines the specific impact of the digital economy on urban TFP using a panel data model with a sample of 285 Chinese prefecture-level cities from 2011 to 2019. Additionally, it employs a mediation effect model to test the mechanisms through which the digital economy influences urban TFP and utilizes a spatial Durbin model to analyze the spatial spillover effects of the digital economy on urban TFP. The research findings reveal the following key points: (1)The digital economy has an overall significantly positive impact on urban TFP. (2)The digital economy indirectly promotes urban TFP by encouraging the advancement of industrial structure and fostering innovation and entrepreneurship in cities. (3)The influence of the digital economy on urban TFP exhibits spatial spillover effects, where the digital economy in neighboring cities significantly enhances the TFP growth of local cities.The results of this study contribute to elucidating the mechanistic pathways through which the digital economy affects urban TFP, holding significant practical implications for achieving high-quality economic development in urban areas.

## Introduction

In 2017, President Xi Jinping emphasized the need for robust development of the digital economy and the integration of digital virtual technologies with the real economy. Leveraging innovative technologies such as information networks, big data, and intelligent cloud computing, China's digital economy has experienced rapid growth in various sectors, including traditional consumer payments, personal credit management, and advanced digital product manufacturing. The scale of the digital economy in China has been steadily expanding, with a report from the China Academy of Information and Communications Technology in 2020 estimating its size at approximately $5.19 trillion in 2019, ranking second globally.

The digital economy has shown remarkable growth momentum, extensive reach, and profound impact. In the current context of China's economic transformation and the continuous growth of the digital economy, leveraging digitalization has become a crucial driver for enhancing total factor productivity, promoting high-quality development, boosting consumption, driving investment, fostering innovation, creating employment, and expediting the digital transformation of production factors. On one hand, the deep integration and penetration of digital technologies into traditional industries have reduced average industry costs through characteristics like decreasing marginal costs, strong dissemination power, and easy circulation, resulting in economies of scale and enhancing total factor output^1^. On the other hand, the features of the digital economy have increased the availability of effective information, facilitating better matching of supply and demand, guiding rational resource allocation, and enhancing the operational efficiency of economic development^2^.

Given these factors, the role of the digital economy as a powerful tool for improving total factor productivity needs to be thoroughly examined and demonstrated. If the answer is affirmative, understanding its mechanisms and whether spatial spillover effects exist becomes crucial. This represents an urgent and essential area of research and inquiry.

The neoclassical growth theory posits that technological progress is a crucial driver of economic development. Technological advancement reflects the efficiency of input factors and output, commonly known as total factor productivity (TFP)^3^. A higher TFP implies that total output is greater for the same level of input factors^4^. Currently, in comparison to developed countries, China's TFP is relatively lower, indicating significant room for improvement^5^.

This study employs a dataset encompassing 285 cities at the prefecture level in China. It utilizes the DEA-Malmquist index model to assess the total factor productivity (TFP) of these cities. Furthermore, it conducts empirical analyses using panel data models to explore the mechanisms and impact pathways through which the digital economy influences urban TFP. Additionally, a spatial Durbin model is constructed to investigate the specific spatial spillover effects of the digital economy on urban TFP.

The research faces three main challenges: Firstly, accurately measuring the development of digital economy at the prefectural level presents a significant challenge. Unlike the wealth of data available at the provincial level, data at the prefectural level is relatively scarce. In this study, we chose to base our analysis on the Urban Digital Inclusive Finance Index published by Peking University. This index was supplemented with indicators related to urban informatization and digital development to depict the level of digital economic development in prefectural cities.

Secondly, there are two primary methods for calculating Total Factor Productivity (TFP): Stochastic Frontier Analysis (SFA) and Data Envelopment Analysis (DEA). Due to the unequal resource endowments among various cities, the DEA-Malmquist productivity index model was chosen to avoid measurement errors associated with model assumptions. This approach was employed to calculate the TFP of 285 prefectural-level cities.

Thirdly, there may exist a bidirectional causality between urban TFP changes and the level of digital economic development. To address endogeneity issues in our research, appropriate instrumental variables must be selected. While existing studies on the digital economy often use data related to postal service quantity from the 1980s as instrumental variables, these studies primarily focus on the provincial level. Many of the prefectural-level cities studied here have shorter histories as cities and lack relevant postal service data. Considering that the development of the digital economy requires financial support, this study chose the reciprocal of the geographical distance to economically developed cities as the instrumental variable.

This study makes three main contributions: (1) Expanded Dataset: The use of data from 285 prefectural-level cities across China significantly expands the sample size and provides a more detailed examination of the research subject. This approach helps address issues related to spurious regression and regional development imbalances. (2) Addressing Endogeneity: The study acknowledges the potential endogeneity issues between the development of the digital economy and total factor productivity (TFP). It employs geographic distance as an instrumental variable and utilizes the Two-Stage Least Squares (2SLS) method to tackle endogeneity concerns effectively. (3) Mechanism Exploration and Spatial Analysis: The research delves into the mechanisms by which the digital economy influences urban TFP and constructs a spatial Durbin model to further investigate its spatial spillover effects. This offers insights into how digital economic development impacts the productivity of neighboring cities.

The remaining sections of this paper are organized as follows: Section “Literature review” presents a comprehensive review of the relevant literature. Section “Research methods and data sources” introduces the research methods, related models, variable selection, and data sources. Section “Empirical analysis and testing” presents the main research findings. Section “Spatial spillover effect analysis” investigated the spatial spillover effects of the digital economy on urban total factor productivity. Section “Discussion” discusses the research findings. Section “Conclusion and recommendations” concludes with policy recommendations.

## Literature review

With the rapid development of the digital economy, its impact on the Total Factor Productivity (TFP) of cities has increasingly become a focal point of research. The digital economy is no longer merely a manifestation of the digitization of production processes; it represents a profound socio-economic transformation, redefining the landscape of socio-economics. The effect of the digital economy on TFP has sparked vigorous debate in academic circles. Current research findings primarily concentrate on the following two aspects.

### Research on the impact of the digital economy on urban total factor productivity

Many scholars agree that the development of the digital economy, reliant on the deep integration of information technology with the traditional industrial economy, has effectively spurred technological advancement and efficiency improvements, thereby steadily enhancing Total Factor Productivity (TFP). Some posited that in urban development, the digital economy is not just a transformation of technological methods but also a driving force for economic structural change, exerting profound and complex effects on urban development. In their study^6^, some utilized panel data from 207 prefecture-level cities and employed a generalized Difference in Differences (DID) model. They focused on national-level big data comprehensive experimental zones as quasi-natural experiments and found that the establishment of these zones significantly boosts urban TFP^7^.

However, the impact of the digital economy on Total Factor Productivity (TFP) remains a subject of debate. Some argues that due to the lack of necessary human and material resources, less developed regions struggle to break the market monopolies established by areas leading in the digital economy. This leads to a pattern of latecomer disadvantage in China's regional growth^8^. Some in their empirical study indicate that the central and western regions, due to a lack of resource endowments and inherent deficiencies in industrial foundations and innovation capabilities, not only find it difficult to enhance TFP through the digital economy, but may even experience a suppression in productivity^9^. Others suggest that the development of the digital economy accentuates the siphoning effect in the southeastern coastal regions, thereby widening the TFP gap on either side of China's Hu Huanyong Line in the short term^10^.

### Study on the mechanisms of digital economy's impact on urban total factor productivity

As understanding of the digital economy deepens and research findings accumulate, the focus of inquiry has gradually shifted from exploring the causal relationship between the digital economy and Total Factor Productivity (TFP) to investigating the mechanisms through which the digital economy affects TFP.

Zhao Wei employed the System GMM method to examine the mechanism by which the digital economy enhances Total Factor Productivity (TFP) through promoting technological innovation capabilities^11^. Similarly, a research demonstrated that the development of the digital economy strengthens environmental regulation, thereby boosting urban TFP. Ji Xueqiang et al. used a panel data model to explore the impact of the digital economy on urban housing prices, finding that the digital economy exerts a negative regulatory effect on the role of housing prices in suppressing TFP^12^. Zhao Wei and Xu Youwen investigated the mechanisms of the digital economy's impact on 110 cities in the Yangtze River Economic Belt from the perspectives of technological innovation efficiency and industrial structure upgrading^13^. The study by Zou Jing et al. also confirmed that industrial structure upgrading is a key mechanism through which the digital economy enhances TFP^14^. Wen Fengan specifically examined the influence pathway between the two from the perspective of factor allocation efficiency^15^. Wang Qiaoran focused on urban agglomerations, finding that the digital economy can promote marketization and thus enhance TFP in urban clusters^16^.

In summary, scholars have conducted relatively extensive research on Total Factor Productivity (TFP), providing valuable insights for reference. However, there are still some issues that require further investigation:

Firstly, few existing studies consider the endogeneity issues present in research. Given the potential bidirectional causality between the digital economy and Total Factor Productivity (TFP), it is imperative to address endogeneity using instrumental variables.

Secondly, there is a lack of in-depth exploration into the spatial spillover effects of the digital economy on urban TFP. The development of the digital economy has facilitated the flow of information and knowledge, leading to the agglomeration of key industries and services. Current research, such as Zhang Yan's study using the Spatial Durbin Model to investigate the impact of provincial digital economy development on TFP^17^, leaves a gap at the urban level.

Thirdly, most analyses on impact mechanisms focus on technological innovation and industrial upgrading. Considering the broad impact of the digital economy on cities, it remains essential to explore other pathways through which it influences urban TFP.

To address these gaps, this paper employs instrumental variables to resolve endogeneity issues in the article. It investigates the impact of the digital economy on urban TFP and its potential mechanisms. Furthermore, it establishes a Spatial Durbin Model to study the spatial spillover effects of the digital economy on urban TFP, which could provide new evidence for assessing the influence of the digital economy on urban TFP.

## Research methods and data sources

### DEA-malmquist index model

#### Data envelopment analysis (DEA)

There are typically two main methods for measuring Total Factor Productivity (TFP), which serve as approaches to assess the efficiency and productivity of economic entities. One approach is Stochastic Frontier Analysis (SFA), introduced by Battese in 1992. SFA models improve the relationship between estimation methods and production functions while avoiding strict data requirements. The second method for calculating TFP is Data Envelopment Analysis (DEA), which evaluates the relative efficiency of production units using linear programming. DEA assesses input–output variables for each unit to gauge their relative efficiency. SFA models rely on determining the specific form of the production function in advance^18^. For the 285 metropolitan areas studied in this paper, there are significant differences in development among regions. For example, some cities are rich in natural resources, while others may have relatively scarce resources. Therefore, assuming that all cities share the same production function is unrealistic. Additionally, since the time span of this study for individual cities is only 9 years, which is relatively short, and the sample size is limited, it does not meet the requirements of SFA models for large sample sizes.

DEA (Data Envelopment Analysis) is a systematic analytical method used to evaluate relative efficiency based on changes in input and output variables of decision-making units. In this context, the decision-making units are represented by metropolitan areas, and it is assumed that there are K metropolitan areas, each with L input indicators and M output indicators. Let's break down the mathematical representation of DEA for each decision-making unit (metropolitan area): $x_{j1}$ represents the input quantity of the first resource for the jth metropolitan area. $y_{jm}$ represents the output quantity of the mth resource for the jth metropolitan area. Where: j = 1, 2, …, K (indicating the K metropolitan areas), l = 1, 2, …, L (representing the L input indicators), m = 1, 2, …, M (representing the M output indicators).

Under the assumptions of convexity, cone, inefficiency, and minimality axioms, DEA is applied to assess the relative efficiency of each metropolitan area in terms of resource inputs and output production. These axioms help ensure that the DEA analysis is carried out effectively and consistently across the different decision-making units:

1 $$\begin{aligned} & \min [\theta - \varepsilon (\hat{e}^{T} s^{ - } + e^{T} s^{ + } )] \\ & s.t.\sum\limits_{j = 1}^{k} {x_{jl} \lambda_{j} + s^{ - } = \theta x_{l}^{n} ;} \sum\limits_{j = 1}^{k} {y_{jm} \lambda_{j} - s^{ + } = y_{m}^{n} } \\ & \lambda_{j} \ge 0;s^{ - } ,s^{ + } \ge 0;n = 1,2, \ldots ,K \\ \end{aligned}$$

In Eq. (1), the variables are defined as follows: $\theta (0 < \theta \le 1)$ represents the comprehensive efficiency index of the metropolitan area, $\lambda_{{\text{j}}}$ denotes the weight variables, $s^{ - }$ represents the slack variables, $s^{ + }$ signifies the residual variables.

#### Malmquist productivity index model

Fare et al. introduced the Malmquist Productivity Index with Constant Returns to Scale (CRS) for assessing changes in productivity from period t to t + 1^19^, as depicted in Eq. (2):

2 $$M\left( {x^{t} ,y^{t} ,x^{t + !} ,y^{t + 1} } \right) = \sqrt {\frac{{D^{t} (x^{t + 1} ,y^{t + 1} )}}{{D^{t} (x^{t} ,y^{t} )}}} \times \sqrt {\frac{{D^{t + !} (x^{t + 1} ,y^{t + 1} )}}{{D^{t + !} (x^{t} ,y^{t} )}}}$$

Using metropolitan areas as an illustration, $D^{t} (x^{t} ,y^{t} )$ and $D^{t} (x^{t + 1} ,y^{t + 1} )$ denote the distance functions between metropolitan areas in periods t and t + 1, with technology from the t-period serving as a reference.

The DEA-Malmquist Index model serves to reduce measurement errors stemming from model specification problems, thus offering a partial remedy to the shortcomings of traditional DEA models. Additionally, the DEA-Malmquist Index model operates independently of the specific production functions of decision-making units, requiring only input and output variables to compute results.

### Model construction

In order to examine the influence of the digital economy on urban total factor productivity, this paper constructs a panel data model, as shown in Eq. (3):

3 $$LnTFP_{it} = \alpha + \beta_{0} *LnDE_{it} + \beta_{1} *LnRSP_{it} + \beta_{2} *LnAIS_{it} + \beta_{3} *LnpGDP_{it} + \beta_{4} *LnIE_{it} + SE_{it} + \delta_{t} + \theta_{c} + \varepsilon_{it}$$

In the equation, $LnTFP_{it}$ represents the natural logarithm of total factor productivity (TFP) for metropolitan areas, $LnDE_{it}$ denotes the natural logarithm of the digital economic development level for metropolitan areas, $LnRSP_{it}$ is the natural logarithm of the indicator measuring the rationalization of industrial structure for metropolitan areas, $LnAIS_{it}$ stands for the natural logarithm of the indicator measuring the advancement of industrial structure for metropolitan areas, $LnpGDP_{it}$ and $LnIE_{it}$ respectively represent the natural logarithms of the economic development level and the innovation and entrepreneurship indicator for metropolitan areas. Taking logarithms serves two purposes: firstly, it reduces the scale differences between variables and helps prevent heteroscedasticity.

The second reason for taking logarithms is to better elucidate the elasticity coefficients of digital economic development on total factor productivity. $SE_{it}$ represents the proportion of scientific education expenditure, calculated as a ratio without logarithmic transformation. $\delta_{t}$ stands for time-fixed effects, $\theta_{c}$ denotes individual fixed effects, and $\varepsilon_{it}$ represents the error term. In this context, "i" represents metropolitan areas, and "t" represents years.

### Variable selection and explanation

#### The dependent variable: total factor productivity (TFP)

This paper utilizes the DEA-Malmquist Index model to compute the Total Factor Productivity (TFP) of 285 metropolitan areas. The output variable is the Gross Domestic Product (GDP) of these metropolitan areas, while the input variables include capital stock and labor force. To adjust the GDP of metropolitan areas for price changes, the base year is set as 2000. However, since the adjusted index for metropolitan areas is not available in the statistical yearbook, this study adopts the adjusted indices for various provinces, as provided by the National Bureau of Statistics, and applies them to the respective metropolitan areas as the adjusted GDP indices.

To calculate the capital stock in metropolitan areas, a depreciation rate of 9.6% is assumed, drawing from the methodology presented by Zhang Jun^20^. When employing the perpetual inventory method to determine capital stock, it is crucial to establish the initial-period capital stock, denoted as *I*_*0*_. In accordance with the methodology^21^, the initial-period capital stock *I*_*0*_ is calculated using the growth rate method, as specified in Eq. (4):

4 $$K_{0} = I_{0} /(g + \delta )$$

In this context, *K*_*0*,_ *I*_*0*_, *g*, and* δ* denote the initial capital stock, capital investment, investment growth rate, and capital depreciation rate, respectively. Assuming steady-state growth, the capital-output ratio remains constant, and in Eq. (8), the investment growth rate "*g*" can be substituted with the GDP growth rate. To determine the base-period capital stock, the average growth rate of fixed asset investment over the five-year period centered around 2003 (i.e., 1998–2008) is used as "*g*" in Eq. (4), facilitating the calculation of capital stock for each metropolitan area.

Presently, the employment data provided by the Chinese National Bureau of Statistics exclusively accounts for urban residents' employment, omitting rural residents' employment from the statistics. Many scholarly references use urban statistical yearbooks' employment figures as a surrogate for labor input. Nevertheless, this approach fails to acknowledge the contributions of informally employed residents to economic progress. Rural residents partake in urban economic endeavors through temporary roles like itinerant vendors and seasonal agricultural laborers, yet they remain unaccounted for in official statistics. Moreover, various metropolitan areas host informal economic activities in which residents generate value through non-formal employment arrangements. These activities bolster overall output growth. Neglecting these aspects and relying on urban residents' employment as a labor proxy can lead to an overestimation of labor input, undermining result accuracy. To address this concern, we adopt the year-end total population of each metropolitan area as a labor proxy, aiming for improved total factor productivity estimation.

#### The primary explanatory variable: digital economy (DE)

Drawing from the research methodology^22^, this study utilizes the City Digital Economy Development Index. This index is based on data published by the Digital Finance Center at Peking University and incorporates various indicators, including telecommunications industry revenue, the number of professionals in the software and computer sector, postal service revenue, year-end mobile phone users, and broadband internet access users for each city. The Entropy method is applied to compute the City Digital Economy Development Index, following standardization of all indicators. The inclusion of postal service revenue is mainly to account for residents in rural and remote areas who often choose postal services as their delivery method for online shopping. These residents' online shopping activities are considered part of the digital economy.

#### Control variable: rationalization of industrial structure (RSP)

The rationalization of industrial structure (RSP) primarily manifests in the balanced development of labor and output proportions among various industries^23^. RSP essentially reflects the output levels of the primary, secondary, and tertiary industries of a city under the same employed workforce. "Rational" implies that, for the labor output of any industry, when compared to the labor output of other industries, it should be approximately equal. Drawing from the research^24^, we calculated the industrial structure rationalization index for each prefecture-level city. A smaller RSP value indicates a more rational industrial structure in the region, while a larger RSP value indicates a less rational industrial structure. The specific expression is as shown in Eq. (5):

5 $$RSP = \sum\nolimits_{i = 1}^{n} {\frac{{Y_{i} }}{Y}\ln \frac{{\frac{{Y_{i} }}{{L_{i} }}}}{\frac{Y}{L}}}$$

Here, $Y_{i} (i = 1,2,3)$ represents the economic output of the primary, secondary, and tertiary industries in the prefecture-level city, using the value-added of the primary, secondary, and tertiary industries of the city to indicate. $L_{i} (i = 1,2,3)$ represents the labor input of the primary, secondary, and tertiary industries in the prefecture-level city. *Y* and *L* respectively denote the total output and total labor input of the prefecture-level city.

Industrial structure upgradation (AIS) refers to the transformation and development of the industrial structure from lower-level forms (such as agriculture, forestry, animal husbandry, and fishing) to middle and high-end forms (such as manufacturing and services). Drawing from the research^25^, it is measured by the ratio of the value-added of the tertiary industry to the value-added of the secondary industry in the city.

Economic development level (PGDP) in this study is represented by the per capita GDP of the prefecture-level city.

The level of urban innovation and entrepreneurship (IE) is represented using the Urban Innovation and Entrepreneurship Index compiled by Peking University's Enterprise Big Data Research Center.

Government support (SE) is measured using the proportion of government spending on science and education relative to total fiscal expenditure in each city.

### The data sources

This study utilizes data from Chinese prefecture-level cities spanning the years 2011 to 2019, resulting in a sample that encompasses 285 cities and a total of 2565 observations. The choice of 2011 as the starting year for the sample period is due to the availability of data on the Digital Inclusive Finance Index, which first appeared in 2011. Data for the control variables are sourced from the "China Urban Statistical Yearbook" as well as individual prefecture-level city statistical yearbooks. In cases where certain cities have missing data, interpolation methods have been applied to complete the dataset.

## Empirical analysis and testing

### The baseline regression analysis

Drawing inspiration from the research approach^26^, to preliminarily examine the impact of digital economic development (DE) on total factor productivity (TFP) at the city level, this study initially employs Ordinary Least Squares (OLS) regression on the model constructed in Eq. (2). The regression results are presented in Table 1.

**Table 1:** Baseline regression results.

Variables	(1)	(2)	(3)
*LnDE*_*it*_	0.183*** (15.08)	0.282*** (25.20)	0.043** (2.10)
*LnRSP*_*it*_	–	− 0.013** (− 2.48)	− 0.019*** (− 3.98)
*LnAIS*_*it*_	–	0.006 (0.45)	0.038** (− 2.39)
*LnPGDP*_*it*_	–	0.255*** (17.17)	0.233*** (9.38)
*LnIE*_*it*_	–	0.071*** (6.72)	0.008 (0.87)
*SE*_*it*_	–	0.001 (0.07)	0.007*** (4.97)
Time fixed effects	No	Yes	Yes
Regional fixed effects	No	No	Yes
*N*	2565	2565	2565
*R*^*2*^	0.101	0.279	0.869

*^,^**^,^***Denote significance levels of 10%, 5%, and 1%, respectively, with t-values shown in parentheses.

In Table 1, the first column presents the explanatory variables along with the dependent variable. The regression analysis reveals that the elasticity coefficient, which measures the responsiveness of urban total factor productivity to changes in digital economic development, is 0.183. This implies that a 1% increase in digital economic development corresponds to a 0.183% rise in urban total factor productivity. Moreover, this elasticity coefficient is statistically significant at the 1% level. This statistical significance underscores the substantial role of digital economic development in enhancing urban total factor productivity.

As shown in the second column of Table 1, when time fixed effects are incorporated, the regression results still indicate a significantly positive relationship. This suggests that over time, the positive impact of digital economic development on urban total factor productivity remains robust. In the third column, after including all control variables and accounting for individual fixed effects, which may include factors such as demographic variations and regional economic policies, the results consistently demonstrate a significant positive influence of digital economic development on urban total factor productivity. These findings reinforce the crucial role of digital economic growth in driving productivity improvements in urban areas.

### Results from instrumental variable regression

Our baseline regression results reveal that the urban digital economy significantly influences urban total factor productivity. However, this relationship might be bidirectional, as higher regional total factor productivity levels could also boost the digital economy's development. Such bidirectional causality introduces endogeneity concerns in our model, potentially distorting the results. To address this, our study adopts an instrumental variables approach. This method helps us more accurately determine the urban digital economy's impact on total factor productivity, thus enhancing the robustness of our findings.

In existing literature, many studies have used the number of post offices or postal service revenue from 1984 as instrumental variables for the digital economy. However, for this research, due to the large-scale implementation of the county-to-city conversion policy in China only starting in the 1990s, many prefecture-level cities had not been established by 1984, leading to a lack of data in this regard. Considering that the development of the digital economy relies on digital equipment, and the expenses incurred in purchasing this digital equipment are significant for both individuals and businesses, the construction of China's digital economy primarily expanded from economically developed regions in the East to relatively less developed regions in the West, and from coastal areas to inland regions. Based on this characteristic, we selected four economically developed prefecture-level cities in the East: Beijing, Shanghai, Hangzhou, and Shenzhen, and used the reciprocal of the average straight-line distance from the remaining 281 prefecture-level cities to these four cities as instrumental variables. The specific calculation formula is shown in Eq. (6):

6 $$IV = \frac{1}{{\frac{1}{4}\sum\limits_{{{\text{i}} = 1}}^{4} {\overline{{d_{i} }} } }}$$

Here, *d*_*i*_ represents the distance between the remaining 281 prefecture-level cities and the four selected prefecture-level cities. Clearly, IV is highly correlated with the digital economy development of prefecture-level cities and unrelated to their economic development level. A larger IV indicates a higher level of digital economy development in the region, while a smaller IV indicates a lower level of digital economy development in the region.

We initially conducted an endogeneity test to evaluate the model. The test results significantly indicated endogeneity, leading us to reject the null hypothesis of no endogeneity at a 1% significance level. We have omitted these detailed results from this paper due to space constraints.

To tackle the identified endogeneity, we used the two-stage least squares (2SLS) method with instrumental variables for re-estimating the regression. This method suits our model's bidirectional causality well. Table 2 presents the outcomes of this instrumental variable regression. The analysis of Table 2 shows that the regression maintains a significant positive effect even after we replace the digital economic development indicator with instrumental variables. These findings strongly validate the baseline regression's conclusions, confirming the significant impact of digital economic development on urban total factor productivity.

**Table 2:** Instrumental variable regression results.

Variables	(1)	(2)	(3)
*LnDE*_*it*_	0.190*** (4.02)	0.191*** (25.08)	0.159*** (4.03)
*LnRSP*_*it*_	–	–	− 0.019*** (− 3.29)
*LnAIS*_*it*_	–	–	0.076*** (5.31)
*LnPGDP*_*it*_	–	–	0.328*** (21.02)
*LnIE*_*it*_	–	–	0.010 (0.98)
*SE*_*it*_	–	–	0.006*** (3.40)
Time fixed effects	No	Yes	Yes
Regional fixed effects	No	No	Yes
*N*	2565	2565	2565
*R*^*2*^	0.110	0.279	0.184

*^,^**^,^***Denote significance levels of 10%, 5%, and 1%, respectively, with t-values shown in parentheses.

### Instrumental variable robustness test

In this section, we further validate the baseline regression's conclusions using alternative instrumental variables. To construct these, we use the 2005 telecommunications revenue of prefecture-level cities as a proxy for the level of digital economic development. Our study, starting from 2011, assumes that the dependent variable, total factor productivity, does not affect the 2005 telecommunications revenue. Yet, a strong correlation exists between telecommunications revenue and digital economic development. Our estimations show that changing the instrumental variables does not alter the significance level or the direction of the coefficient representing the digital economy's impact on urban total factor productivity. This consistency suggests that our earlier conclusions are both robust and reliable.

### Heterogeneity analysis

#### Regional heterogeneity analysis

China, with its vast expanse and regional disparities in development, presents an ideal setting to examine the impact of digital economic development on total factor productivity across different prefecture-level cities. To address these regional disparities, we have categorized our research sample into three main regions: the Eastern region, the Central region, and the Western region. The regression results are presented in Table 3.

**Table 3:** Regional heterogeneity regression results.

Variables	Eastern region	Central region	Western region
*LnDE*_*it*_	0.247*** (11.96)	0.123*** (3.15)	0.147*** (4.63)
*LnRSP*_*it*_	− 0.014** (− 2.50)	− 0.049*** (− 7.29)	− 0.025*** (− 4.79)
*LnAIS*_*it*_	0.011 (0.77)	0.062*** (3.47)	0.099*** (5.01)
*LnPGDP*_*it*_	0.249*** (15.98)	0.179*** (9.76)	0.227*** (8.11)
*LnIE*_*it*_	0.055*** (4.99)	0.028** (2.24)	0.018* (1.82)
*SE*_*it*_	0.001 (0.35)	0.008*** (4.15)	0.004** (2.35)
Time fixed effects	Yes	Yes	Yes
Regional fixed effects	Yes	Yes	Yes
*N*	909	1026	630
*R*^*2*^	0.461	0.873	0.431

*^,^**^,^***Denote significance levels of 10%, 5%, and 1%, respectively, with t-values shown in parentheses.

Table 3 clearly demonstrates that digital economic development positively and significantly affects the total factor productivity of cities across all three regions. Most notably, the Eastern region shows the highest positive impact, followed by the Western region. The Central region, however, shows the smallest effect. Comparing these results with the nationwide regression for all cities, digital economic development significantly influences the Eastern region more than the national average. Conversely, the Central and Western regions slightly fall behind the national average in this respect.

Several factors can explain these findings. The Eastern region, with its coastal proximity, enjoys natural geographical advantages. These advantages promote efficient transportation, foster large-scale economic activities, and attract talent, potentially leading to a 'Matthew effect' where the strong get stronger. In this region, digital economic development directly boosts total factor productivity, thereby enhancing economic output. In contrast, the Western region, grappling with greater economic challenges and lower total factor productivity compared to the Central region, sees a more pronounced impact of digital economic development on its total factor productivity. This difference highlights how regional characteristics can influence the efficacy of digital economic development in boosting productivity.

These regional variations underscore the importance of considering geographic disparities when analyzing the relationship between digital economic development and urban productivity, as they provide valuable insights into the complex dynamics at play within different regions of China.

#### Urban size heterogeneity analysis

Population size plays a crucial role in influencing urban development. Larger populations correlate positively with a larger potential labor force, as cities with more people typically have access to more human capital, both in physical and intellectual labor. Additionally, cities with larger populations often possess greater consumption capacity, benefiting economic output. Moreover, population size impacts not just a city's total factor productivity but also its level of digital economic development.

In this study, we categorized cities based on population size to examine whether the impact of digital economic development on total factor productivity varies with different urban sizes. Specifically, we classified cities as large cities if their year-end population exceeded five million, small cities if their population was less than one million, and medium-sized cities if their population fell between one million and five million.

Table 4's regression results show that digital economic development positively promotes total factor productivity in cities of various sizes. However, in small cities, this impact is not statistically significant and fails hypothesis testing. A likely explanation is the lack of adequate infrastructure in smaller cities, particularly infrastructure necessary for digital economic development. The results also highlight that in larger cities, digital economic development significantly boosts total factor productivity. This underscores the crucial role of human capital in not only enhancing a city's total factor productivity but also in advancing its digital economic development.

**Table 4:** Heterogeneity analysis by urban size.

Variables	Nationwide	Large cities	Medium-sized cities	Small cities
*LnDE*_*it*_	0.161*** (3.95)	0.275*** (14.34)	0.225*** (7.88)	0.123 (1.33)
*LnRSP*_*it*_	− 0.044*** (− 6.15)	− 0.034*** (− 4.55)	− 0.017*** (2.64)	− 0.023*** (− 4.30)
*LnAIS*_*it*_	0.042** (2.22)	− 0.019 (− 0.95)	0.018 (1.08)	0.126*** (6.19)
*LnPGDP*_*it*_	0.196*** (10.27)	0.210*** (10.51)	0.242*** (14.08)	0.153*** (5.27)
*LnIE*_*it*_	0.026* (1.93)	0.026* (1.79)	0.035** (2.86)	− 0.002 (− 0.20)
*SE*_*it*_	0.008*** (3.99)	0.007** (2.88)	0.002 (1.13)	− 0.001 (− 0.40)
Time fixed effects	Yes	Yes	Yes	Yes
Regional fixed effects	Yes	Yes	Yes	Yes
*N*	2565	891	1557	117
*R*^*2*^	0.178	0.641	0.336	0.864

*^,^**^,^***Denote significance levels of 10%, 5%, and 1%, respectively, with t-values shown in parentheses.

### Mechanism examination

In theoretical terms, the impact pathways of the digital economy on urban total factor productivity (TFP) can be manifested through the rationalization of industrial structure, the upgradation of industrial structure, and the promotion of urban innovation and entrepreneurship. Regarding industries, the development of the digital economy contributes to the digitization and digital transformation of industries. Digital production methods not only accelerate capital circulation and enhance the efficiency of capital and equipment utilization but also reduce decision-making time, further improving production efficiency.

Furthermore, the development of the digital economy also drives the construction of urban digital infrastructure, including 5G base stations, network bandwidth, and urban cloud services. These digital infrastructures provide fertile ground for urban innovation and entrepreneurship. Innovation and entrepreneurship not only generate new economic output but also serve as a vehicle for the application and dissemination of new technologies. The application of these new technologies in societal contexts contributes to the enhancement of urban total factor productivity. A prime example is Silicon Valley in the United States, where the significant development of high-tech enterprises has notably elevated the region's economic development and technological progress.

Regarding this mechanism, this study employs a mediation effect model to investigate the mechanisms through which digital economic development influences urban total factor productivity. The constructed model is represented by the following equation:

7 $$LnRSP_{it} = \alpha + \beta *LnDE_{it} + \lambda *X_{it} + \delta_{t} + \theta_{c} + \varepsilon_{it}$$

8 $$LnAIS_{it} = \alpha + \beta *LnDE_{it} + \lambda *X_{it} + \delta_{t} + \theta_{c} + \varepsilon_{it}$$

9 $$LnIE_{it} = \alpha + \beta *LnDE_{it} + \lambda *X_{it} + \delta_{t} + \theta_{c} + \varepsilon_{it}$$

In this equation, $LnRSP_{it}$, $LnAIS_{it}$, and $LnIE_{it}$ represent industrial structure rationalization, industrial structure upgradation, and urban innovation and entrepreneurship, respectively. *X*_*it*_ denotes control variables. *δ*_*t*_ represents time fixed effects, *θ*_*i*_ represents regional fixed effects, and *ε*_*it*_ stands for the error term.

Table 5 displays the regression results for the impact of digital economic development on the three variables. The results in Table 5 indicate a non-statistically significant negative regression coefficient of digital economic development on urban industrial structure rationalization, failing to surpass the 10% significance level in hypothesis testing. This lack of statistical significance suggests that digital economic development exerts some degree of promotion on urban industrial structure rationalization. It enhances the upgrading of industrial structure in cities and has a significantly positive impact on urban innovation and entrepreneurship. The mediation analysis results mentioned earlier indicate that digital economic development promotes the improvement of urban total factor productivity by facilitating the upgrading of industrial structure and fostering urban innovation and entrepreneurship.

**Table 5:** Regression results for mediation mechanism.

Variables	The rationalization of industrial structure	The advancement of industrial structure	Urban innovation and entrepreneurship
*LnDE*_*it*_	− 0.036 (− 0.28)	0.105*** (3.77)	0.113** (2.43)
*LnRSP*_*it*_	–	− 0.016** (− 2.50)	− 0.053* (− 1.87)
*LnAIS*_*it*_	− 0.416*** (− 5.37)	–	0.005 (0.48)
*LnPGDP*_*it*_	0.998*** (13.89)	− 0.526*** (− 16.10)	0.344*** (12.12)
*LnIE*_*it*_	− 0.087 (− 1.55)	− 0.014 (− 1.11)	–
*SE*_*it*_	0.058*** (6.70)	0.002 (1.09)	0.036*** (10.92)
Time fixed Effects	Yes	Yes	Yes
Regional fixed effects	Yes	Yes	Yes
*N*	2565	2565	2565
*R*^*2*^	0.837	0.890	0.887

*^,^**^,^***Denote significance levels of 10%, 5%, and 1%, respectively, with t-values shown in parentheses.

### Spatial spillover effect analysis

Cities are strongly interconnected economically, and the economic development of one city can influence the economic development of neighboring cities. Generally, cities that are closer to each other have a more significant mutual impact. Tobler proposed the "First Law of Geography," which suggests that the degree of connection between things is related to distance^27^. This principle also applies to the mutual influence of cities on each other's total factor productivity. In the era of the digital economy, innovation and entrepreneurship platforms exhibit digital characteristics, characterized by networked and information-based features. The digital development model breaks down spatial barriers to information exchange and dissemination, significantly promoting information sharing and production exchange among neighboring regions. Network platforms connect various economic activities such as research and development, design, production, sales, supply chain logistics, and provide highly dispersed technical support markets for innovation activities, facilitating cross-regional innovation cooperation in different fields. In this development process, spatial spillover phenomena occur, meaning that the development of the digital economy in one region can affect the total factor productivity of other cities. In the previous models, we assumed that there was no spatial correlation among error terms, which could lead to biased estimation results. To address spatial spillover effects, we use spatial econometric methods to further investigate the relationship between the digital economy and urban total factor productivity.

### The spatial Durbin model construction

The spatial Durbin model combines the characteristics of the spatial error model (SEM) and the spatial lag model (SAR) and can analyze regional differences and spatial patterns. This is of great significance for studying the development of TFP in different cities under the influence of the digital economy. Through spatial analysis of this model, it is possible to identify the advantages and potential of specific regions, providing support for urban development and resource allocation.

Drawing inspiration from the research^28^, we have constructed a Spatial Panel Durbin Model to investigate the impact of digital economy development on urban total factor productivity (TFP). The model is represented as Eq. (10):

10 $$LNTFP_{it} = \rho W_{ij} LNTFP_{it} + \beta_{1} LnDE_{it} + \gamma X_{it} + W_{ij} (\beta_{3} LnDE_{it} + \varepsilon X_{it} ) + \mu_{i}$$

whereas, ${X}_{it}$ represents the control variables. ${u}_{i}$ signifies the spatial fixed effects. ${W}_{ij}$ stands for the weight matrix. *i* denotes different cities. *t* represents different years.

The spatial econometric model requires the design of a spatial weight matrix to reflect the spatial impact of neighboring areas on the local area. To demonstrate the robustness of spatial estimation results, this paper considers constructing both economic-geographic distance spatial weight matrix and economic distance spatial weight matrix from economic and geographical distance perspectives, respectively. The economic-geographic distance weight matrix (W_1_) constructed in this paper is shown in Eq. (11):

11 $$\begin{aligned} W_{1,ij} & = W_{d} diag(\overline{{Y_{1} }} /\overline{Y} ,\overline{{Y_{2} }} /\overline{Y} , \cdots \overline{{Y_{n} }} /\overline{Y} ) \\ W_{d} & = \left\{ \begin{gathered} \frac{1}{{d_{ij} }} \cdots d_{ij} \ge d \hfill \\ 0 \cdots d_{ij} \le d \hfill \\ \end{gathered} \right. \\ \end{aligned}$$

where ${W}_{d}$ represents the inverse of the straight-line Euclidean distance (${d}_{ij}$) between prefecture-level cities, used to construct the first-order inverse distance geographic weight matrix. $\overline{{Y_{i} }} = \frac{1}{{t_{n} - t_{0} + 1}}\sum\nolimits_{{t_{0} }}^{{t_{n} }} {Y_{it} }$ represents the annual average GDP of prefecture-level city i during the observation period, and $\overline{Y}$ represents the annual average GDP of all regions during the observation period.

The expression for constructing the economic distance weight matrix (W_2_) in this study is as shown in Eq. (12):

12 $$W_{2.ij} = \left\{ \begin{gathered} \frac{1}{{\overline{{Y_{i} }} - \overline{{Y_{j} }} }} \cdots i = j \hfill \\ 0 \cdots i = j \hfill \\ \end{gathered} \right.$$

where: $\overline{{Y_{i} }} = \frac{1}{{t_{n} - t_{0} + 1}}\sum\nolimits_{{t_{0} }}^{{t_{n} }} {Y_{it} }$ represents the average regional GDP of prefecture-level city *i* during the observation period. $\overline{{Y_{{\text{j}}} }} = \frac{1}{{t_{n} - t_{0} + 1}}\sum\nolimits_{{t_{0} }}^{{t_{n} }} {Y_{{{\text{j}}t}} }$ represents the average regional GDP of prefecture-level city *j* during the observation period.

Previous studies have indicated that using point estimates to test for spatial spillover effects in spatial panel models can lead to bias. Therefore, it is necessary to decompose the parameter vector in the spatial Durbin model into direct and indirect effects using a partial differentiation approach^29^. The vector form of the spatial Durbin model can be represented as follows:

13 $$Y_{t} = (I_{n} - \rho W)^{ - 1} (X_{t} \beta + WX_{t} \theta ) + (I_{n} - \rho W)^{ - 1} \varepsilon_{t}^{*}$$

The error term comprises random errors, spatial effects, and time effects. It represents the change in the dependent variable relative to the k-th explanatory variable for different spatial units at any specified time. The partial differentiation matrix of (${x}_{ik}$, $i=1, 2\dots , N$) is as follows:

14 $$\left[ {\frac{\partial Y}{{\partial X_{1k} }},\frac{\partial Y}{{\partial X_{2k} }}...\frac{\partial Y}{{\partial X_{Nk} }}} \right]_{t} = \left[ \begin{gathered} \frac{{\partial y_{1} }}{{\partial X_{1k} }}\mathop {}\limits^{{}} \frac{{\partial y_{1} }}{{\partial X_{2k} }}...\frac{{\partial y_{1} }}{{\partial X_{Nk} }} \hfill \\ \frac{{\partial y_{2} }}{{\partial X_{1k} }}\mathop {}\limits^{{}} \frac{{\partial y_{2} }}{{\partial X_{2k} }}...\frac{{\partial y_{2} }}{{\partial X_{Nk} }} \hfill \\ \mathop {}\limits_{{}} ...\mathop {}\limits_{{}} \mathop {}\limits_{{}} \mathop {}\limits_{{}} \mathop {}\limits_{{}} ...\mathop {}\limits_{{}} \mathop {}\limits_{{}} \mathop {}\limits_{{}} \mathop {}\limits_{{}} ... \hfill \\ \frac{{\partial y_{N} }}{{\partial X_{1k} }}\mathop {}\limits^{{}} \frac{{\partial y_{N} }}{{\partial X_{2k} }}...\frac{{\partial y_{N} }}{{\partial X_{Nk} }} \hfill \\ \end{gathered} \right] = (I_{n} - \rho W)\left[ \begin{gathered} \mathop {}\limits^{{}} \beta_{k} \mathop {}\limits^{{}} \mathop {}\limits^{{}} w_{12} \theta_{k} ...w_{1N} \theta_{k} \hfill \\ w_{21} \theta_{k} \mathop {}\limits^{{}} \mathop {}\limits^{{}} \beta_{k} \mathop {}\limits^{{}} ...w_{2N} \theta_{k} \hfill \\ \mathop {}\limits_{{}} ...\mathop {}\limits_{{}} \mathop {}\limits_{{}} \mathop {}\limits_{{}} \mathop {}\limits_{{}} ...\mathop {}\limits_{{}} \mathop {}\limits_{{}} \mathop {}\limits_{{}} \mathop {}\limits_{{}} ... \hfill \\ w_{N1} \theta_{k} \mathop {}\limits^{{}} w_{N2} \beta_{k} ...\beta_{k} \hfill \\ \end{gathered} \right]$$

In the above equation, the direct effect is the average of the elements on the diagonal of the partial differentiation matrix on the right-hand side, while the indirect effect is the average of the non-diagonal elements corresponding to the rows or columns of that matrix.

### Spatial correlation test

The prerequisite for using a spatial econometric model is the presence of spatial correlation among individual units. In this study, we employ the Global Moran's I to test for spatial autocorrelation. The Moran's I statistic is calculated as shown in Eq. (15):

15 $$I = \frac{{n\sum_{i = 1}^{n} \sum_{j = 1}^{n} \omega_{ij} (x_{i} - \overline{x} )(x_{j} - \overline{x} )}}{{(\sum_{i = 1}^{n} \sum_{j = 1}^{n} \omega_{ij} )\sum_{i = 1}^{n} (x_{i} - \overline{x} )^{2} }}$$

Here, ${x}_{i}$ represents the study subjects in various cities; *n* is the number of regions (spatial units); $\overline{x} = \frac{{\sum_{i = 1}^{n} x_{i} }}{n}$; $\omega$ denotes the spatial weight matrix; and *I* takes values within the range of [− 1, 1].

This study performed a Moran's test on urban total factor productivity and digital economic development levels using two distinct weight matrices. The results of the spatial autocorrelation tests are detailed in Table 6. Both total factor productivity and digital economic development have exhibited significance in the Moran's test at the 1% level, indicating a robust spatial interaction effect among different cities concerning these variables. This finding remains consistent across various spatial matrices, reinforcing the robustness of spatial correlation. Traditional research methods assume independence among variables across different cities, a bias that is evident. Given the outcomes of the Moran's test, it is imperative to consider spatial dependency among cross-sectional units. Consequently, employing spatial econometric models becomes essential for accurately estimating the spatial effects of digital economic development on urban total factor productivity.

**Table 6:** Moran's I test results.

Year	Total factor productivity (TFP)				Development of the digital economy			
	Matrix W_1_		Matrix W_2_		Matrix W_1_		Matrix W_2_	
	Moran's I	P-value	Moran's I	P-value	Moran's I	P-value	Moran's I	P-value
2011	0.163	0.000	0.096	0.000	0.280	0.000	0.152	0.000
2012	0.193	0.000	0.115	0.000	0.283	0.000	0.151	0.000
2013	0.232	0.000	0.132	0.000	0.237	0.000	0.147	0.000
2014	0.226	0.000	0.141	0.000	0.259	0.000	0.138	0.000
2015	0.221	0.000	0.140	0.000	0.257	0.000	0.135	0.000
2016	0.184	0.000	0.140	0.000	0.250	0.000	0.123	0.000
2017	0.166	0.000	0.141	0.000	0.276	0.000	0.134	0.000
2018	0.172	0.000	0.137	0.000	0.265	0.000	0.138	0.000
2019	0.165	0.000	0.140	0.000	0.263	0.000	0.135	0.000

### Selection of spatial panel model

LM tests indicate that utilizing spatial econometric models is more appropriate than non-spatial econometric models. Both the Wald_SDM/SAR and LR_SDM/SAR values have successfully passed a significant test at the 1% level, rejecting the null hypothesis that the spatial Durbin model can degenerate into a spatial autoregressive model. Similarly, the Wald_SDM/SEM and LR_SDM/SEM values have also passed a significant test at the 1% level, rejecting the null hypothesis that the spatial Durbin model can degenerate into a spatial error model. Consequently, this paper adopts the spatial Durbin model, which simultaneously accounts for time and research individuals, as the empirical model. The test results are presented in Table 7, focusing solely on the economic geography distance weight matrix (W_1_) and economic distance weight matrix (W_2_).

**Table 7:** Test of applicability of space Durbin model.

Test type	W_1_	W_2_
LM test		
LM_error	153.543***	576.253***
Robust_LM_error	55.701***	190.968***
LM_lag	264.450***	543.753***
Robust_LM lag	151.820***	167.136***
LR test		
LR_SDM/SAR	76.988***	82.988***
LR_SDM/SEM	78.689***	70.224***
Wald test		
Wald_SDM/SAR	33.458***	51.630***
Wald_SDM/SEM	83.408***	75.213***
Fixed effects test		
LR_both/ind	80.191***	79.455***
LR_both/time	563.388***	469.876***

*^,^**^,^***Denote significance levels of 10%, 5%, and 1%, respectively, with t-values shown in parentheses.

### Analysis of spatial Durbin model estimation results

Empirical results are presented in Table 8. To ensure the robustness of our findings, we conducted regression analyses using two different spatial matrices. Columns (1) and (2) present estimations using the economic-geographic distance weight matrix (W_1_), while columns (3) and (4) employ the economic distance weight matrix (W_2_). Columns (1) and (3) do not consider any control variables, while columns (2) and (4) include all control variables in the analysis.

**Table 8:** Spatial Durbin model estimation results.

Variables	Economic geographic distance weight matrix (W_1_)		Economic distance weight matrix (W_2_)	
	(1)	(2)	(3)	(4)
*LnDE*_*it*_	0.168*** (9.74)	0.173*** (9.36)	0.239*** (12.44)	0.205*** (12.11)
*LnRSP*_*it*_	–	− 0.142**(− 2.62)	–	− 0.015** (− 2.76)
*LnAIS*_*it*_	–	− 0.006 (− 0.42)	–	0.006 (0.40)
*LnPGDP*_*it*_	–	0.238*** (15.32)	–	0.236*** (14.58)
*LnIE*_*it*_	–	0.062*** (4.92)	–	0.045*** (3.52)
*SE*_*it*_	–	0.001 (0.09)	–	0.008 (0.49)
W*LnDE_*it*_	0.155*** (4.77)	0.257*** (5.73)	0.156*** (3.92)	0.191*** (3.95)
W*LnRSP_*it*_	–	0.008 (0.52)	–	− 0.006 (− 0.40)
W*LnAIS_*it*_	–	− 0.035 (− 0.91)	–	0.108*** (2.99)
W*LnPGDP_*it*_	–	− 0.015 (− 0.39)	–	− 0.083** (− 2.32)
W*LnIE_*it*_	–	0.095*** (3.63)	–	0.130*** (− 5.51)
W*SE_*it*_	–	− 0.001 (− 0.16)	–	− 0.002 (− 0.54)
*Spatial-rho*	0.181*** (4.66)	0.156*** (4.07)	0.446*** (15.01)	0.356*** (11.11)
*sigma2_e*	0.103*** (33.90)	0.087*** (33.92)	0.012*** (33.37)	0.082*** (33.43)
Time fixed effects	Yes	Yes	Yes	Yes
*R*^2^	0.345	0.321	0.344	0.367
Sample size	2565	2565	2565	2565

*^,^**^,^***Denote significance levels of 10%, 5%, and 1%, respectively, with t-values shown in parentheses.

The regression results in columns (1) and (3) demonstrate significance at the 1% confidence level, implying the presence of a "spillover effect" in the development of the digital economy in this region. This effect indicates that the progress of the digital economy not only fosters the enhancement of total factor productivity within the local area but also plays a role in augmenting total factor productivity in neighboring regions.

The results in columns (2) and (4) demonstrate the following.

Even after accounting for control variables, the coefficient representing the influence of the digital economy on local total factor productivity remains positive and statistically significant at the 1% level. This suggests that the development of the local digital economy has a substantial positive impact on local total factor productivity. The digital economy contributes not only to the promotion of industrial restructuring and optimization through the digitization and digitalization of industries but also facilitates urban innovation and entrepreneurship. By providing both software and hardware support to startups, it contributes to an increase in local total factor productivity.

Secondly, the significant enhancement of total factor productivity in neighboring cities due to local digital economy development is noteworthy. This phenomenon can be attributed to the inherent characteristics of the digital economy, characterized by its foundational, integrative, and pervasive nature. These features make it highly conducive to fostering cross-regional economic collaboration.

These findings underscore the pivotal role of the digital economy, not only in augmenting local productivity but also in facilitating economic cooperation among neighboring regions.

To further assess the impact of digital economic development on total factor productivity, following the methodology of LeSage and Pace, we decompose the impact into direct effects, indirect effects, and total effects. The results of the spatial Durbin model regression for digital economic development and total factor productivity are presented in Table 9.

**Table 9:** Effects of digital economic development on total factor productivity.

Variables	Economic geographic distance weight matrix (W_1_)			Economic distance weight matrix (W_2_)		
	Direct effect	Indirect effect	Total effect	Direct effect	Indirect effect	Total effect
*LnDE*_*it*_	0.178*** (10.56)	0.463*** (6.92)	0.641*** (9.89)	0.211*** (11.72)	0.261*** (4.79)	0.472*** (8.98)
Control variables	Yes	Yes	Yes	Yes	Yes	Yes
Time fixed effects	Yes	Yes	Yes	Yes	Yes	Yes
R^2^	0.323			0.362		
Sample size	2565					

*^,^**^,^***Denote significance levels of 10%, 5%, and 1%, respectively, with t-values shown in parentheses.

Analyzing Table 9, it is evident that when estimated using the economic-geographic distance weight matrix W_1_, all three effects of digital economic development on urban total factor productivity attain statistical significance at the 1% level. The total effect coefficient for digital economic development stands at 0.641, signifying that, on average, a 1% increase in the level of digital economic development corresponds to approximately a 0.641% increase in urban total factor productivity. The direct effect reveals an estimated coefficient of 0.178, indicating that the local development of the digital economy positively influences local total factor productivity. Specifically, a 1% increase in digital economic development results in a 0.178% increase in total factor productivity. The estimated indirect effect suggests that a 1% increase in digital economic development in the local region leads to a 0.463% increase in total factor productivity in neighboring cities.

When estimating using the economic distance weight matrix W_2_, the three effects of digital economic development on total factor productivity remain statistically significant at the 1% level, and the sign of the coefficients remains consistent, confirming the robustness of the conclusions. The total effect coefficient for digital economic development is 0.472, suggesting that a 1% increase in the level of digital economic development leads to an overall increase of 0.472% in total factor productivity. The direct effect coefficient for digital economic development is 0.211, indicating that local digital economic development positively influences local total factor productivity, with a 1% increase in digital economic development leading to a 0.211% increase in total factor productivity. The estimated indirect effect shows that for every 1% increase in digital economic development in the local region, neighboring regions experience a 0.261% increase in total factor productivity.

In conclusion, digital economic development has a dual impact, promoting both local and neighboring region's total factor productivity growth, as evident from the results of the spatial Durbin model estimations.

## Discussion

With the rapid development and widespread adoption of digital technology, the digital economy has become a new engine for global economic development. The rise of the digital economy has not only transformed traditional production methods and business models but also had profound effects on the overall productivity of cities. In this study, we used data from 285 prefecture-level cities in China and proposed a new instrumental variable to overcome the endogeneity issue in the research. We employed spatial econometric models to analyze the spatial spillover effects of the digital economy.

Different from the studies conducted by Yi et al.^30^ and Wang and Shao^31^, who used the number of post offices in 1984 as an instrumental variable for studying the digital economy, this study employed the reciprocal of the average straight-line distance from the remaining 281 prefecture-level cities to Beijing, Shanghai, Hangzhou, and Shenzhen as the instrumental variable.

The selection of instrumental variables is crucial for accurately estimating the results. In this study, we used different instrumental variables and obtained conclusions consistent with the studies of Yuan^19^ and Yang and Jiang^22^ on the impact of provincial digital economy on overall factor productivity. This validates that the digital economy not only promotes provincial overall factor productivity but also contributes to urban overall factor productivity.

This study found that the digital economy can promote the improvement of urban overall factor productivity. This conclusion is based on a comprehensive evaluation and analysis of the various impacts of the digital economy on urban economies. The innovation-driven role of the digital economy is one of the important factors in promoting the improvement of urban overall factor productivity. Through the application of digital technology, enterprises can enhance production efficiency and quality by conducting production and management more efficiently.

The development of digital economy can have an impact on the regional economic development, thereby promoting the improvement of regional factor productivity^32^. Our research verifies this viewpoint at the urban level. The cross-industry integration feature of digital economy makes the industrial chains between cities more interdependent and interconnected. Different enterprises and organizations in different cities achieve closer cooperation and collaboration through the application of digital technology, thus forming a more efficient economic ecosystem. In addition, the information connectivity feature of digital economy promotes more frequent and direct information exchange between cities, which facilitates the transfer and sharing of knowledge, technology, and management experience among different cities. This cross-city flow of knowledge and resources promotes the economic development of surrounding cities and improves their overall factor productivity.

However, it should be noted that the impact of digital economy on surrounding cities is also subject to certain restrictions and conditions. The "siphon effect" that may be caused by mega-cities on surrounding small cities could have adverse effects on their development. Therefore, in future research, we need to further explore the specific effects of these factors on the cross-city impact of digital economy and propose corresponding policy recommendations to promote a more beneficial pattern of digital economy development.

In our study, we found that the coefficient of the impact of the digital economy on the total factor productivity of Chinese cities is approximately 0.190. This implies that a 1% increase in the digital economy will lead to an approximate 0.190% increase in the total factor productivity of cities. Compared to previous research on the impact of the digital economy on city-level total factor productivity, our findings suggest a smaller effect. This result is attributed to our utilization of instrumental variable methods to address endogeneity concerns, thereby leading us to consider this result as closer to the true scenario.

Due to a lack of relevant statistical data, most studies on the digital economy have chosen a time period starting from 2011. Prior to 2011, although some economically developed cities had already disclosed their digital economy development, these cities were relatively few in number, leading to sample selection bias and an inability to comprehensively consider the influence of various historical factors on the research results. This means that we cannot fully understand and compare the impact of the digital economy on overall urban factor productivity before 2011. In future research, our objective is to further explore the generalizability of our conclusions by utilizing data with longer time spans.

## Conclusion and recommendations

### Conclusion

With the vigorous development of information technology, digital technology has integrated with traditional financial services, giving rise to the emergence of the new format of digital finance. Urban total factor productivity reflects a city's ability to integrate technology, resources, and management. Studying the impact of digital finance on total factor productivity is essentially an investigation into the influence of digital technology and financial elements on integration capabilities. China's vast territory results in significant disparities in development and resource endowments among its cities. Digital finance, to some extent, reduces the reliance on physical production factors and compensates for the imbalances in resource distribution among cities, providing a more equitable starting point for competition.

This study, based on panel data from 285 prefecture-level cities in China from 2011 to 2019, employs panel data models to investigate the impact of the digital economy on total factor productivity. It uses a mediation effects model to examine the underlying mechanisms and employs a spatial Durbin model to analyze the spatial spillover effects of the digital economy on urban total factor productivity. The study explores the impact mechanisms and effects of these two factors. The research findings are as follows:

1. In summary, the digital economy significantly enhances urban total factor productivity. Moreover, even after addressing endogeneity issues with instrumental variables, the positive impact of digital economy development on urban total factor productivity remains statistically significant.
2. Taking regional heterogeneity into consideration, the study identifies that the digital economy exerts the most substantial positive impact on total factor productivity in eastern cities, followed by western cities, while its effect is comparatively weaker in central cities. Further analysis based on population heterogeneity indicates that in cities with larger populations, the local digital economy has a more pronounced positive impact on total factor productivity.
3. The investigation into the impact mechanisms of the digital economy on urban total factor productivity reveals that it fosters the rationalization and sophistication of urban industrial structures while positively influencing urban innovation and entrepreneurship. Through these two pathways, the digital economy enhances urban total factor productivity.
4. Utilizing a spatial Durbin model, the study uncovers spatial spillover effects in the impact of the digital economy on urban total factor productivity. This means that the development of the digital economy in one area not only enhances local total factor productivity but also significantly promotes the total factor productivity of neighboring cities.

### Recommendations

First, promote the development of the digital economy and its related industries. Develop comprehensive plans for industries related to the digital economy, including the telecommunications industry, computer hardware technology industry, software industry, internet industry, artificial intelligence industry, big data application industry, and digital manufacturing industry. Establish and improve the ecosystem of industries related to the digital economy. Enhance the development of digital infrastructure by accelerating the deployment of 5G base stations and building supporting internet hardware facilities, laying a solid hardware foundation for the digital economy's growth. Increase financial and fiscal support for the digital economy and its related industries to provide funding for their upgrading.

Second, tailor digital economy development strategies to local conditions, taking into account the specific circumstances of each city's development. Prioritize a people-centric approach and give preference to technological advancements, focusing on advancing digital industrialization and industrial digitization. In eastern cities, leverage their existing economic development levels and advantageous geographic locations by launching talent attraction initiatives, improving incentives for digital talent, and relaxing residency restrictions for professionals. In central cities, expedite the transformation of traditional industries towards digitization and informatization, fostering sustained growth of digital industries. In western cities, formulate development plans for digital economy-related infrastructure, establish clusters of digital economy industries, and create centers for intelligent manufacturing.

Third, harness the spatial spillover effects of the digital economy and pay attention to disparities in the development of different cities while promoting coordinated urban development. Foster cross-regional collaboration through means such as talent exchanges, product markets, and the development of factors of production to achieve complementary cooperation across regions. Encourage the formation of cross-regional digital alliances among digital economy-related enterprises and establish digital development platforms to facilitate the growth of the digital economy in various regions.

## Data Availability

The datasets collected and analyzed during the current study are not publicly available due to issues related to authorship and copyright but are available from the corresponding author on reasonable request. If you indeed require access to the original data, please contact eshaozsh@xust.edu.cn.
